# Occluded Superior Mesenteric Artery and Vein. Therapy with the Stable Gastric Pentadecapeptide BPC 157

**DOI:** 10.3390/biomedicines9070792

**Published:** 2021-07-08

**Authors:** Mario Knezevic, Slaven Gojkovic, Ivan Krezic, Helena Zizek, Dominik Malekinusic, Borna Vrdoljak, Tamara Knezevic, Hrvoje Vranes, Domagoj Drmic, Miro Staroveski, Antonija Djuzel, Zoran Rajkovic, Toni Kolak, Eva Lovric, Marija Milavic, Suncana Sikiric, Ante Tvrdeic, Leonardo Patrlj, Sanja Strbe, Marija Sola, Andrej Situm, Antonio Kokot, Alenka Boban Blagaic, Anita Skrtic, Sven Seiwerth, Predrag Sikiric

**Affiliations:** 1 Department of Pharmacology, School of Medicine, University of Zagreb, 10000 Zagreb, Croatia; mariknezevic@gmail.com (M.K.); slaven.gojkovic.007@gmail.com (S.G.); ivankrezic94@gmail.com (I.K.); zizekhelena@gmail.com (H.Z.); dominikmalekinusic@gmail.com (D.M.); borna.vrdoljak@gmail.com (B.V.); 0101tamara@gmail.com (T.K.); hrvoje.vranes@gmail.com (H.V.); iddrmic@mef.hr (D.D.); miro.staroveski@gmail.com (M.S.); antonija.djuzel@gmail.com (A.D.); tkolak@kbd.hr (T.K.); ante.tvrdeic@mef.hr (A.T.); patrljleo@gmail.com (L.P.); strbes@gmail.com (S.S.); marijasola11@gmail.com (M.S.); andrej_situm@yahoo.com (A.S.); abblagaic@mef.hr (A.B.B.); 2 Department of Surgery, Faculty of Dental Medicine and Health, University of Osijek, 31000 Osijek, Croatia; zrajkovi@net.hr; 3 Department of Pathology, School of Medicine, University of Zagreb, 10000 Zagreb, Croatia; eva.lovric@kb-merkur.hr (E.L.); marija.milavic@mef.hr (M.M.); suncanasikiric@gmail.com (S.S.); seiwerth@mef.hr (S.S.); 4 Department of Anatomy and Neuroscience, Faculty of Medicine Osijek, J. J. Strossmayer University of Osijek, 31000 Osijek, Croatia; antonio.kokot@mefos.hr

**Keywords:** BPC 157, superior mesenteric vein and artery occlusion, vascular recruitment, rats

## Abstract

Background. We investigated the occluded essential vessel tributaries, both arterial and venous, occluded superior mesenteric vein and artery in rats, consequent noxious syndrome, peripherally and centrally. As therapy, we hypothesized the rapidly activated alternative bypassing pathways, arterial and venous, and the stable gastric pentadecapeptide BPC 157 since it rapidly alleviated venous occlusion syndromes. Methods. Assessments were performed for 30 min (gross recording, venography, ECG, pressure, microscopy, biochemistry, and oxidative stress), including portal hypertension, caval hypertension, aortal hypotension, and centrally, the superior sagittal sinus hypertension; systemic arterial and venous thrombosis, ECG disturbances, MDA-tissue increase, the multiple organs lesions, heart, lung, liver, kidney and gastrointestinal tract, including brain (swelling, and cortex (cerebral, cerebellar), hypothalamus/thalamus, hippocampus lesions). Rats received BPC 157 medication (10 µg/kg, 10 ng/kg) intraperitoneally at 1 min ligation-time. Results. BPC 157 rapidly activated collateral pathways. These collateral loops were the superior mesenteric vein-inferior anterior pancreaticoduodenal vein-superior anterior pancreaticoduodenal vein-pyloric vein-portal vein pathway, an alternative pathway toward inferior caval vein via the united middle colic vein and inferior mesenteric vein through the left colic vein, and the inferior anterior pancreaticoduodenal artery and inferior mesenteric artery. Consequently, BPC 157 counteracted the superior sagittal sinus, portal and caval hypertension, aortal hypotension, progressing venous and arterial thrombosis peripherally and centrally, ECG disturbances attenuated. Markedly, the multiple organs lesions, heart, lung, liver, kidney, and gastrointestinal tract, in particular, as well as brain lesions, and oxidative stress in tissues were attenuated. Conclusions. BPC 157 therapy rapidly recovered rats, which have complete occlusion of the superior mesenteric vein and artery.

## 1. Introduction

Simultaneous occlusion of the superior mesenteric artery and vein is a rare but serious complication and has recently been reported in COVID-19 patients [1]. While the regular therapy includes fluid resuscitation, analgesia, anticoagulation, broad-spectrum antibiotics, and emergency surgery [1,2], we investigated a novel approach—rapid activation of the collateral pathways [3,4]—to overcome the occluded superior mesenteric vein and artery and counteract the consequent syndrome in rats. The stable gastric pentadecapeptide BPC 157 has been shown to alleviate venous occlusion syndromes—inferior caval vein syndrome [5], Pringle manoeuvre ischemia/reperfusion [6], and Budd Chiari syndrome [7]—rapidly activating alternative bypassing pathways. These bypassing loops—the left ovarian vein [5], the inferior mesenteric vein [6], and the azygos vein [7]—are reliant on the corresponding injurious occlusion [5,6,7] to re-establish blood flow that compensates for vessel occlusion. BPC 157 therapy would likely represent a ‘bypassing key’ [5,6,7], rapidly activating bypassing pathways and abrogating the complex syndrome induced by simultaneous occlusion of essential arterial and venous tributaries.

Studies have shown that BPC 157 led to activation of the alternative collateral pathways to bypass occlusion and re-established alternative blood flow that counteracts the negative consequences of the perilous syndromes [5,6,7]. The severe venous occlusion-induced disturbances [5,6,7], the high portal and caval hypertension, aortal hypotension, arterial and venous thrombosis, and lesions of peripheral and central organs (i.e., gastrointestinal system, liver, kidney heart, and brain) are all attenuated and/or eliminated [5,6,7]. Furthermore, these beneficial effects may compete with Virchow’s triad, which could be present [5,6,7,8,9,10,11,12]. In addition to the mentioned syndromes [5,6,7], BPC 157 provided beneficial effects in the duodenal venous congestion lesions [8], perforated cecum [9], damaged peritoneum [10], ischemia/reperfusion colitis [11], and bile duct ligation-induced liver cirrhosis and portal hypertension [12].

Importantly for the large superior mesenteric artery and vein tributary, BPC 157 benefitted the entire gastrointestinal tract, has a very safe profile (the lethal dose (LD1) could not be achieved [13]) and has shown efficacy in ulcerative colitis trials [3,4,14,15]. Overall, it combines its particular mediating role in Robert’s stomach cytoprotection [3,16,17,18] as a unique anti-ulcer peptide that is stable in human gastric juice for more than 24 h [19], distinctive from the standard angiogenic growth factors that are rapidly degraded in human gastric juice [13,19]. Conceptually, agents that provide Robert’s cytoprotection against direct epithelial cell injury due to direct contact of various noxious agents [17] must protect the endothelium and maintain its function [18]. BPC 157 fulfils these criteria in the entire gastrointestinal tract [3,4,13,14,15], maintains stomach endothelium integrity against noxious agents [20], prevents and reverses thrombosis formation after abdominal aorta anastomosis [21] or major vein occlusion [5,6,7], and maintains thrombocyte function without interfering with coagulation pathways [22,23,24]. Based on the therapeutic effects of BPC 157 in venous occlusion studies [5,6,7], this peptide provides endothelium maintenance and promotes blood vessel recruitment and activation (‘running’) towards the site of injury, also described as ‘bypassing’ the occlusion via alternative pathways [3,4]. Regarding arterial occlusion, there is evidence that BPC 157 counteracts stroke when given during reperfusion, after clamping of the common carotid arteries (i.e., both early and delayed neural hippocampal damage, achieving full functional recovery) [25]. BPC 157 appears to play a role in the brain–gut axis and has central effects [26], and BPC 157 administration can also counteract central disturbances (i.e., brain swelling, intracranial hypertension), which may likely appear in the rats subjected to permanent, simultaneous occlusion of the superior mesenteric vein and artery (i.e., increased intra-abdominal pressure and increased intracranial pressure) [27]. Hence, BPC 157 represents a treatment to drain venous blood adequately from the brain without raising venous pressures. In this way, this peptide ‘bypasses’ the occlusion [3,4] to overcome the permanent, simultaneous occlusion (ligation) of the superior mesenteric vein and artery and counteracts the consequent deadly syndrome.

The nitric oxide (NO) system also plays a crucial role in the effects of BPC 157 on the endothelium [28]. BPC 157 regulates vasomotor tone through specific activation of the Src–caveolin-1–endothelial nitric oxide synthase (eNOS) pathway [29], and it has a modulatory role [3,4,28] for blood pressure [30] and maintaining thrombocyte function [22,23,24]. BPC 157 also interacts with several molecular pathways [5,25,29,31,32,33,34,35,36,37] and may be a free radical scavenger to counteract reperfusion-induced oxidative injury [5,6,8,9,10,11,12,36,37,38,39,40]. BPC 157 acts as a membrane stabiliser, counteracting leaky gut syndrome by interacting with several molecular pathways [37]; thereby, its curative effect involves modulating ischemia-induced increased capillary permeability [37].

When the end of the superior mesenteric vein is occluded, the superior mesenteric vein–inferior anterior pancreaticoduodenal vein–superior anterior pancreaticoduodenal vein–pyloric vein–portal vein pathway may be essential to re-establish the superior mesenteric vein and portal vein connection and blood flow. Providing, that in rats, caudally, the united are tributaries of the superior and inferior mesenteric vein, an alternative pathway toward the inferior caval vein may be via the united middle colic vein and inferior mesenteric vein through its left branch. Furthermore, considering superior mesenteric artery occlusion in rats, the inferior anterior pancreaticoduodenal artery and the inferior mesenteric artery should be simultaneously prone to provide rapid bypassing and vessel recruitment. Studies have shown that BPC 157 therapy was effective in rats in which the superior anterior pancreaticoduodenal vein was occluded [8] as well as in the rats with simultaneous occlusion of the left colic artery and vein [11]. In those studies, BPC 157 counteracted duodenal lesions [8] and ischemia/reperfusion colitis [11].

Based on the reported benefits of BPC 157, we examined the effect of therapy with this peptide on the syndrome produced due to simultaneous occlusion of the superior mesenteric vein and artery. Specifically, we examined portal and inferior caval vein hypertension; abdominal aorta hypotension; superior sagittal sinus hypertension, and thereby rapid brain swelling and extensive severe brain lesions, leading to progressing venous and arterial thrombosis; electrocardiogram (ECG) disturbances; and lesions in the lungs, liver, kidneys, gastrointestinal tract, and brain. We used the same dose range (µg–ng BPC 157/kg body weight) that had been tested in animal models of venous occlusion [5,6,7]) and found the BPC 157 served as a ‘bypassing key’ and promoted beneficial effects to counteract simultaneous occlusion of the superior mesenteric artery and vein.

## 2. Materials and Methods

### 2.1. Animals

This study was conducted with 12-week-old male albino Wistar rats (200 g) randomly assigned to different groups (6 rats/group/interval). Rats were bred in-house at the Pharmacology Animal Facility, School of Medicine, Zagreb, Croatia. The animal facility has been registered by the Directorate of Veterinary (Reg. No: HR-POK-007). Laboratory rats were acclimated for 5 days and randomly assigned to their respective treatment groups. Laboratory animals were housed in polycarbonate (PC) cages under conventional laboratory conditions at 20–24 °C, relative humidity of 40–70% and a noise level of 60 dB. Each cage was identified with dates, the study number, the group number, the dose, and the number of each animal. Fluorescent lighting provided illumination 12 h per day. A standard good laboratory practice (GLP) diet and fresh water were provided ad libitum. Animal care was in compliance with the standard operating procedures (SOPs) of the Pharmacology Animal Facility and the European Convention for the Protection of Vertebrate Animals used for Experimental and Other Scientific Purposes (ETS 123).

This study was approved by the local Ethics Committee. The study complied with the European Directive 010/63/E, the Law on Amendments to the Animal Protection Act (Official Gazette 37/13), the Animal Protection Act (Official Gazette 135/06), the ordinance on the protection of animals used for scientific purposes (Official Gazette 55/13), the Federation of European Laboratory Animal Science Associations (FELASA) recommendations, and the recommendations of the Ethics Committee of the School of Medicine, University of Zagreb. The experimental results were assessed by observers blinded to the treatment of each animal.

### 2.2. Drugs

The stable gastric pentadecapeptide BPC 157 is a partial sequence of the human gastric juice protein BPC, which is freely soluble in water at pH 7.0 and in saline. BPC 157 (GEPPPGKPADDAGLV, molecular weight 1419; Diagen, Ljubljana, Slovenia) was prepared as a peptide with 99% high-performance liquid chromatography (HPLC) purity, with the 1-des-Gly peptide being the main impurity. The dose and application regimens were as described previously; it was administered without the use of a carrier or a peptidase inhibitor [5,6,7,8,9,10,11,12].

### 2.3. Experimental Protocol

In deeply anesthetised rats—intraperitoneal [ip] injection of 40 mg/kg thiopental (Rotexmedica, Trittau, Germany) and 10 mg/kg diazepam (Apaurin; Krka, Novo Mesto, Slovenia)—we simultaneously completely occluded the end of the superior mesenteric vein (ligation) just below the joining of the lienal vein as well as close to abdominal aorta the complete occlusion of the superior mesenteric artery. Thereby, permanent occlusion leads to permanent alteration of arterial and venous blood flow and a progressive disease course.

Treatments were given intraperitoneally (1 mL/rat via an abdominal bath) at 1 min after ligation: 10 µg/kg BPC 157, 10 ng/kg BPC 157 or 5 mL/kg saline. All rats were sacrificed 30 min after ligation.

For venography, the treatments (10 µg/kg BPC 157, 10 ng/kg BPC 157 or 5 mL/kg saline) were applied intraperitoneally, as 1 mL/rat via an abdominal bath, 15 min after ligation, just before venography.

Brain swelling was recorded in rats 15 min after the complete calvariectomy was performed. Briefly, six burr holes were drilled in three horizontal lines, all of them medial to the superior temporal lines and temporalis muscle attachments. The two rostral burr holes were placed just basal from the posterior interocular line, the two basal burr holes were placed just rostral to the lambdoid suture (and transverse sinuses) on both sides, respectively) and the middle two burr holes were placed in the line between the basal and rostral burr holes.

Rats were laparatomized for the corresponding presentation of the peripheral veins (superior mesenteric, inferior mesenteric, inferior anterior pancreaticoduodenal, jejunal, middle colic, left colic, portal and inferior caval) and arteries (superior mesenteric artery, proximal and distal to occlusion, inferior mesenteric artery, abdominal aorta). The recording was with a camera attached to a VMS-004 Discovery Deluxe USB microscope (Veho, Dayton, OH, USA) performed until the end of the experiment, and assessed at 5, 15, and 30 min after ligation.

### 2.4. Venography

Venography was performed 15 min after ligation of the superior mesenteric vein and artery, using a C-VISION PLUS fluoroscopy unit (Shimadzu, Chiyoda, Tokyo, Japan) [18,19,20]. One millilitre of warmed Omnipaque 350 (iohexol) non-ionic contrast medium (GE Healthcare, Arlington Heights, IL, USA) was injected over 45 s into the superior mesenteric vein below the occlusion. The contrast medium was visualised in real-time to ensure adequate filling. A subtraction mode was used to record the images at 14 frames/s. Fifteen minutes after ligation, venograms were captured and digitised into files on a personal computer and were analysed using ISSA image software (Vamstec, Zagreb, Croatia). Venography assessment included rats having a full presentation of collaterals and bypassed occlusion.

### 2.5. Superior Sagittal Sinus, Portal, Superior Mesenteric and Caval Vein and Abdominal Aorta Pressure Recording

As described previously [5,6,7], recordings were made in deeply anesthetised rats with a cannula (BD Neoflon™ Cannula, Eysins, Switzerland) connected to a pressure transducer (78534C MONITOR/ TERMINAL; Hewlett Packard, Houston, TX, USA) inserted into the portal vein, inferior caval vein, and superior sagittal sinus, and abdominal aorta at the level of the bifurcation at 30 min post-ligation after 5 min of recording. For superior sagittal sinus pressure recording, we made a single burr hole in the rostral part of the sagittal suture, above the superior sagittal sinus and cannulated the anterior part of the superior sagittal sinus by Braun intravenous cannulas. We then laparatomized rats for portal vein, inferior vena cava, and abdominal aorta pressure recording.

Notably, normal rats exhibited a superior sagittal sinus pressure of −24 to −27 mmHg and superior mesenteric pressure and portal pressure of 3–5 mmHg, which is similar to that of the inferior vena cava, although with values at least 1 mmHg higher in the portal vein. By contrast, abdominal aorta blood pressure values were 100–120 mmHg at the level of the bifurcation [5,6,7].

### 2.6. ECG Recording

ECGs were recorded continuously in deeply anesthetised rats for all three main leads, by positioning stainless steel electrodes on all four limbs using an ECG monitor with a 2090 programmer (Medtronic, Minneapolis, MN, USA) connected to a Waverunner LT342 digital oscilloscope (LeCroy, Chestnut Ridge, NY, USA) after 30 min of ligation. This arrangement enabled precise recordings, measurements, and analysis of ECG parameters [5,6,7].

### 2.7. Thrombus Assessment

After the rats were euthanized, the superior sagittal sinus, portal vein, external jugular vein, inferior caval vein, superior mesenteric vein, hepatic vein, superior mesenteric artery, and abdominal aorta were removed from the rats, and the clots were weighed [18,19,20].

### 2.8. Brain Volume and Vessel Presentation Proportional to the Change in the Brain or Vessel Surface Area

The presentation of the brain and peripheral veins (superior mesenteric and inferior mesenteric, portal, inferior caval, inferior anterior pancreaticoduodenal, jejunal, middle colic, left colic, and inferior caval) and peripheral arteries (superior mesenteric artery, inferior mesenteric artery, and abdominal aorta) was recorded in deeply anesthetised rats, with a camera attached to a VMS-004 Discovery Deluxe USB microscope (Veho, Dayton, OH, USA). The recordings were performed before the ligation as well as 5, 15, and 30 min after ligation. The border of the brain or veins in a photograph was marked using ImageJ software (National Institutes of Health, Bethesda, MD, USA) and then the surface area (in pixels) of the brain or veins was measured using a measuring function. This procedure was performed using brain photographs before and at different times after the application of saline (control) or BPC 157. The area of the brain, veins, or arteries before the application was marked as 100% and the ratio of each subsequent brain area to the first area was calculated ($\frac{A_{2}}{A_{1}}$). Starting from the square-cube law Equations (1) and (2), an equation for the change in brain volume proportional to the change in the brain surface area (6) was derived. In expressions (1)–(5), l is defined as any arbitrary one-dimensional length of the brain (e.g., rostro-caudal length of the brain), used only for defining one-dimensional proportion (l_2_/l_1_) between two observed brains and as an inter-factor (and because of that not measured) to derive the final expression (6). The procedure was as follows:

(1) $$A_{2}=A_{1}{\left(\frac{l_{2}}{l_{1}}\right)}^{2}\;(\mathrm{square}-\mathrm{cube}\;\mathrm{law});$$

(2) $$V_{2}=V_{1}{\left(\frac{l_{2}}{l_{1}}\right)}^{3}\;(\mathrm{square}-\mathrm{cube}\;\mathrm{law});$$

(3) $$\frac{A_{2}}{A_{1}}={\left(\frac{l_{2}}{l_{1}}\right)}^{2}\;(\mathrm{from}\;(1),\;\mathrm{after}\;\mathrm{dividing}\;\mathrm{both}\;\mathrm{sides}\;\mathrm{by}\;A_{1});$$

(4) $$\frac{l_{2}}{l_{1}}=\sqrt{\frac{A_{2}}{A_{1}}}\;(\mathrm{from}\;(3),\;\mathrm{after}\;\mathrm{taking}\;\mathrm{square}\;\mathrm{root}\;\mathrm{of}\;\mathrm{both}\;\mathrm{sides});$$

(5) $$\frac{V_{2}}{V_{1}}={\left(\frac{l_{2}}{l_{1}}\right)}^{3}\;(\mathrm{from}\;(2),\;\mathrm{after}\;\mathrm{dividing}\;\mathrm{both}\;\mathrm{sides}\;\mathrm{by}\;V_{1});\;\mathrm{and}$$

(6) $$\frac{V_{2}}{V_{1}}={\left(\sqrt{\frac{A_{2}}{A_{1}}}\right)}^{3}\;(\mathrm{after}\;\mathrm{incorporating}\;(4)\;\mathrm{into}\;(5)).$$

### 2.9. Presentation of Gastrointestinal Lesions and Serosal Disturbances

A camera attached to a VMS-004 Discovery Deluxe USB microscope (Veho, Dayton, OH, USA) was used for recording. In deeply anesthetised rats, at 30 min post-ligation, we assessed the gross lesions in the gastrointestinal tract (haemorrhagic congestive areas in the stomach and duodenum, jejunum, cecum, ascending colon, and rectum, calculated as the sum of the longest diameters in millimetres) and serosal disturbances (haemorrhage, vessels ramification, arterial filling, congestion in jejunum, cecum, ascending colon, and rectum). Serosal disturbances were scored 0–4 for several categories. The first was bleeding on the intestinal surface: 0—no bleeding; 1—barely indicated bleeding (diameter of the hematoma on the intestinal surface <1 mm); 2—mild bleeding (diameter of the hematoma on the intestinal surface >1 mm to 2 mm); 3—moderate bleeding (diameter of the hematoma on the intestinal surface >2 mm to 4 mm); 4—intense bleeding (diameter of the hematoma on the intestinal surface >4 mm). Venous congestion included the following categories: 0—venous congestion not present; 1—barely indicated venous congestion (vein thickness up to 0.5 mm); 2—mild venous congestion (vein thickness >0.5 mm to 1 mm); 3—moderate venous congestion (vein thickness >1 mm to 2 mm); 4 intense venous congestion (vein thickness >2 mm to ≥2.5 mm). Arterial filling included the following categories: 0—arterial filling not noticeable; 1—barely indicated arterial filling (artery thickness up to 0.5 mm); 2—mild arterial filling (artery thickness >0.5 mm to 0.75 mm); 3—moderate arterial filling (artery thickness >0.75 mm to 2 mm); 4—intensive arterial filling (artery thickness >2 mm to ≥2.5 mm). Arterial ramification included the following categories: 0—no noticeable ramification; 1—barely indicated arterial ramification (two branches visible); 2—mild arterial ramification (three branches visible); 3—moderate arterial ramification (four branches visible); 4—intensive arterial ramification (≥five branches).

### 2.10. Liver and Spleen Weights

Liver and spleen weights are expressed as a percent of the total body weight (for normal rats, the liver is 3.2–4.0% of the body weight and the spleen is 0.20–0.26% of the body weight).

### 2.11. Bilirubin and Enzyme Activity

To determine the serum levels of aspartate transaminase (AST), alanine transaminase (ALT, IU/L), and total bilirubin (μmol/L), blood samples were collected from the inferior caval vein immediately before euthanasia and were centrifuged for 15 min at 3000 rpm. All tests were performed using an Olympus AU2700 analyser with original test reagents (Olympus Diagnostics, Ireland) [12]. However, the bilirubin data are not shown because there was no change in the level.

### 2.12. Microscopy

The brain, liver, kidney, stomach, duodenum, jejunum, colon, rectum, lungs, and heart were removed 30 min after ligation of the superior mesenteric vein and artery. The tissues were fixed in 10% neutral buffered formalin (pH 7.4) at room temperature for 24 h. Representative tissue specimens were embedded in paraffin, sectioned at 4 μm, stained with haematoxylin and eosin (H&E), and evaluated by light microscopy.

The brain was dissected according to NTP-7 Levels 3 and 6 with neuroanatomic subsites presented in certain brain sections using at least three coronal sections [41]. Fronto-parietal cortex, hippocampus, thalamus, and hypothalamus were observed and analysed at NTP-7 Level 3. At NTP-7 Level 6, the cerebellar cortex morphology was analysed using a semiquantitative neuropathological scoring system from 0 to 8 as described previously (Table 1) [42], where 0 indicates no histopathologic damage.

#### 2.12.1. Lung Histology

A scoring system was used to grade the degree of lung injury. The considered features were focal thickening of the alveolar membranes, congestion, pulmonary oedema, intra-alveolar haemorrhage, interstitial neutrophil infiltration, and intra-alveolar neutrophil infiltration. Each feature was assigned a score from 0 to 3 based on its absence (0) or presence to a mild (1), moderate (2), or severe (3) degree, and a final histology score was determined [43].

#### 2.12.2. Renal, Liver and Heart Histology

The renal injury criteria were based on the degeneration of Bowman’s space and glomeruli, degeneration of the proximal and distal tubules, vascular congestion, and interstitial oedema. The liver injury criteria were vacuolisation of hepatocytes and pyknotic hepatocyte nuclei, activation of Kupffer cells, and enlargement of sinusoids. Each specimen was scored using a scale from 0 to 3 (0—none; 1—mild; 2—moderate; 3—severe) for each criterion, and a final histology score was determined [44].

The heart lesion estimation was based on dilatation and congestion of blood vessels within the myocardium and coronary arteries using a scale from 0 to 3 (0—none; 1—mild; 2—moderate; 3—severe).

#### 2.12.3. Gastrointestinal Histology

Intestinal tissue damage was analysed using a histologic scoring scale adapted from Chui and co-workers [45] on a scale from 0 to 5 (normal to severe) in three categories (mucosal injury, inflammation, hyperaemia/haemorrhage) for a total score of 0 to 15 as described by Lane and co-workers [46]. Morphologic features of mucosal injury were based on different grades of epithelia lifting, villi denudation, and necrosis; grades of inflammation were from focal to diffuse according to lamina propria infiltration or subendothelial infiltration; hyperaemia/haemorrhage was graded from focal to diffuse according to lamina propria or subendothelial localisation.

### 2.13. Oxidative Stress

Thirty minutes after ligation, oxidative stress in the collected tissue samples (plasma, ICV) was assessed by quantifying thiobarbituric acid reactive species (TBARS) as malondialdehyde (MDA) [5,6,38,39,40]. The tissue samples were homogenised in phosphate-buffered saline (PBS, pH 7.4) containing 0.1 mM butylated hydroxytoluene (BHT) (TissueRuptor, Qiagen, Valencia, CA, USA) and sonicated for 30 s in an ultrasonic ice bath (Branson, USA). Trichloroacetic acid (TCA, 10%) was added to the homogenate, the mixture was centrifuged at 3000 rpm for 5 min and the supernatant was collected. Then, 1% TBA was added, and the samples were boiled (95 °C, 60 min). The tubes were then kept on ice for 10 min. Following centrifugation (14,000 rpm, 10 min), the absorbance of the mixture at 532 nm was determined.

The concentration of MDA was determined from a standard calibration curve plotted using 1,1,3,3′-tetrethoxy propane (TEP). The extent of lipid peroxidation is expressed as MDA using a molar extinction coefficient for MDA of 1.56 × 10^5^ mol/L/cm. The protein concentration was determined using a commercial kit. The results are expressed in nmol/g of protein.

### 2.14. Statistical Analysis

Statistical analysis was performed by parametric one-way analysis of variance (ANOVA), with the post hoc Newman–Keuls test, or non-parametric Kruskal–Wallis test, followed by the Mann-Whitney U test to compare groups. Values are presented as the mean ± standard deviation (SD) or as the minimum, median and maximum. To compare the frequency difference between groups, the chi-square test or Fischer’s exact test was used. *p* < 0.05 was considered statistically significant.

## 3. Results

**Figure 1:**
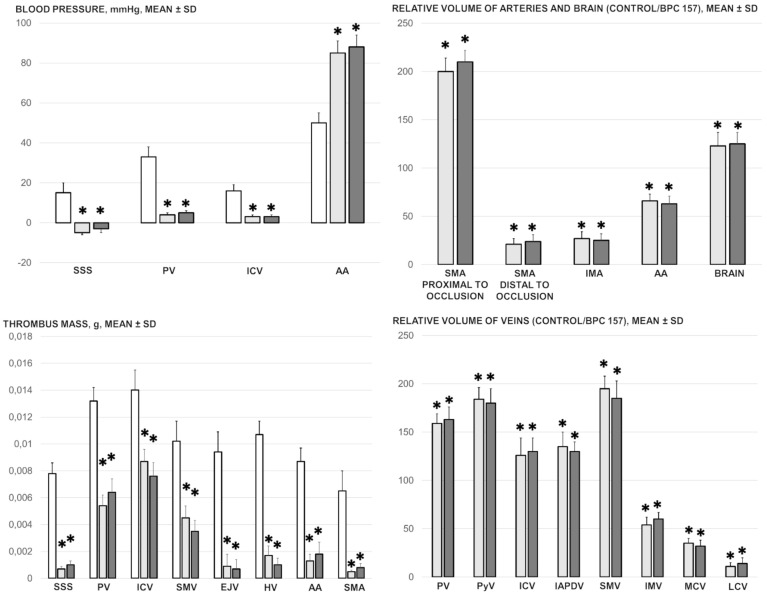
Blood pressure, mm Hg (in the superior sagittal sinus (SSS), portal vein (PV), abdominal aorta (AA), inferior caval vein (ICV)), thrombus mass, g (in the superior sagittal sinus (SSS), portal vein (PV), inferior caval vein (ICV), superior mesenteric vein (SMV), external jugular vein (EJV), hepatic veins (HV), abdominal aorta (AA) and superior mesenteric artery (SMA)), relative volume of arteries (superior mesenteric artery (SMA), proximal to occlusion and distal to occlusion, inferior mesenteric artery (IMA), abdominal aorta (AA) and brain) and relative volume of veins (portal vein (PV), pyloric vein (PyV), inferior caval vein (ICV), inferior anterior pancreaticoduodenal vein (IAPDV), superior mesenteric vein (SMV), inferior mesenteric vein (IMV), middle colic vein (MDV) and left colic vein (LCV)) presentation in the rats with ligated superior mesenteric vein and artery at the 30 min ligation-time, following medication (BPC 157 10 µg/kg (light gray bars), 10 ng/kg (dark gray bars); saline 5 mL/kg (white bars) given intraperitoneally. Especially, as described previously [5,6,7], pressure recordings were made in deeply anesthetised rats with a cannula (BD Neoflon™ Cannula, Franklin Lakes, NJ, USA) connected to a pressure transducer (78534C MONITOR/TERMINAL; Hewlett Packard, Houston, TX, USA) inserted into the portal vein, inferior caval vein and superior sagittal sinus, and abdominal aorta at the level of the bifurcation at 30 min post-ligation after 5 min of recording (see, Section 2.5). Six rats/group/interval. Mean ± SD, * *p* ˂ 0.05, at least vs. control.

**Figure 2:**
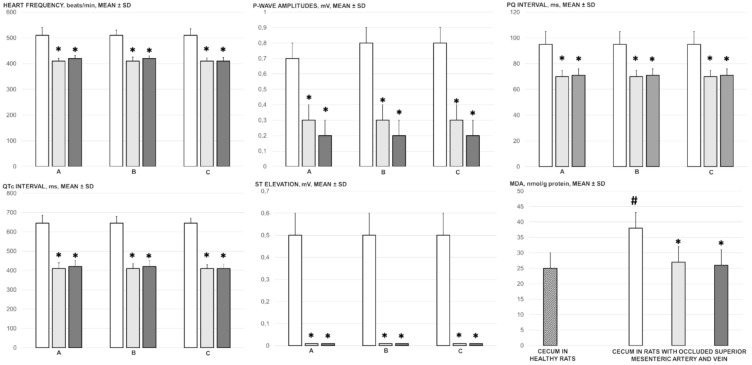
ECG changes (heart frequency (beats/min), P-wave amplitude (mV), PQ interval (ms), QTc interval (ms), ST-elevation (mV)) at 5 min (A), 15 min (B) and 30 min (C) ligation-time, oxidative stress (MDA, nmol/g protein), in the rats with ligated superior mesenteric vein and artery at 30 min ligation-time following medication (BPC 157 10 µg/kg (light gray bars), 10 ng/kg (dark gray bars); saline 5 mL/kg (white bars) given intraperitoneally. Six rats/group/interval. Mean ± SD, * *p* ˂ 0.05, at least vs. control, # *p* ˂ 0.05, at least vs. healthy (dashed bar).

**Figure 3:**
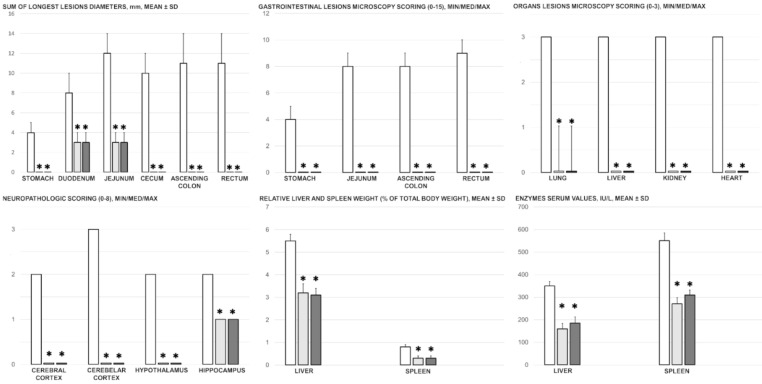
Gross gastrointestinal lesions presentation (sum of the longest lesions diameters, mm, mean ± SD), gastrointestinal lesions microscopy (scoring (0–15, Min/Med/Max), organs (lung, liver, kidney, and heart) lesions microscopy (scoring (0–3, Min/Med/Max), neuropathologic scoring (cerebral cortex, cerebellar cortex, hypothalamus, hippocampus, 0–8), enzymes serum values (IU/L, mean ± SD), liver and spleen relative weight (% of total body weight, mean ± SD) at 30 min ligation-time following medication (BPC 157 10 µg/kg (light gray bars), 10 ng/kg (dark gray bars); saline 5 mL/kg (white bars) given intraperitoneally. Six rats/group/interval. * *p* ˂ 0.05, at least vs. control.

**Figure 4:**
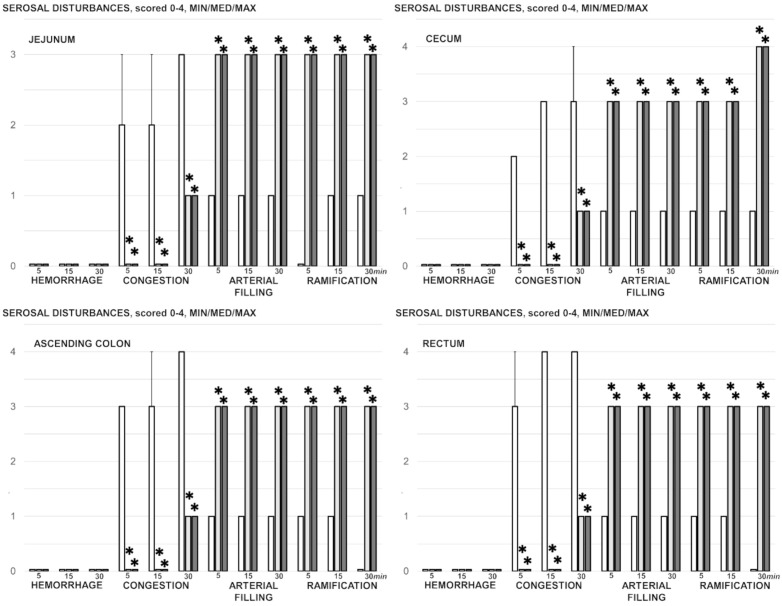
Serosal disturbances (hemorrhage, congestion, arterial filling, ramification), scored 0–4, Min/Med/Max, at 5 min, 15 min and 30 min ligation-time in rats with occluded superior mesenteric vein and artery following medication (BPC 157 10 µg/kg (light gray bars), 10 ng/kg (dark gray bars); saline 5 mL/kg (white bars) given intraperitoneally. Six rats/group/interval. * *p* ˂ 0.05, at least vs. control.

**Figure 5:**
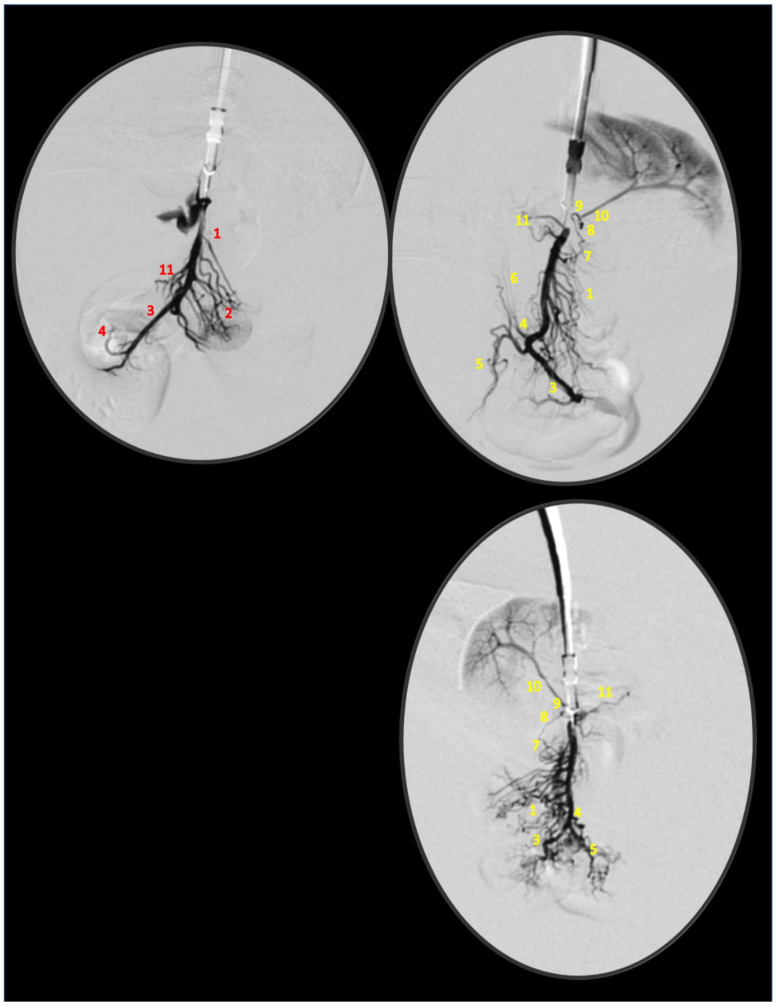
Superior mesenteric vein (below ligation) venography, and veins presentation (jejunal branches of superior mesenteric vein (1), ileal branches of superior mesenteric vein (2), superior mesenteric vein (3), ileocolic/right colic vein truncus (4), ileocolic vein (5), right colic vein (6), inferior anterior pancreaticoduodenal vein (7), superior anterior pancreaticoduodenal vein (8), pyloric vein (9), portal vein (10), middle colic vein (11)) in rats with the occlusion of the superior mesenteric vein and artery immediately following medication (BPC 157 10 µg/kg (upper), or 10 ng/kg (low) (right) or saline 5 mL/kg (left)) given intraperitoneally at 15 min ligation-time.

**Figure 6:**
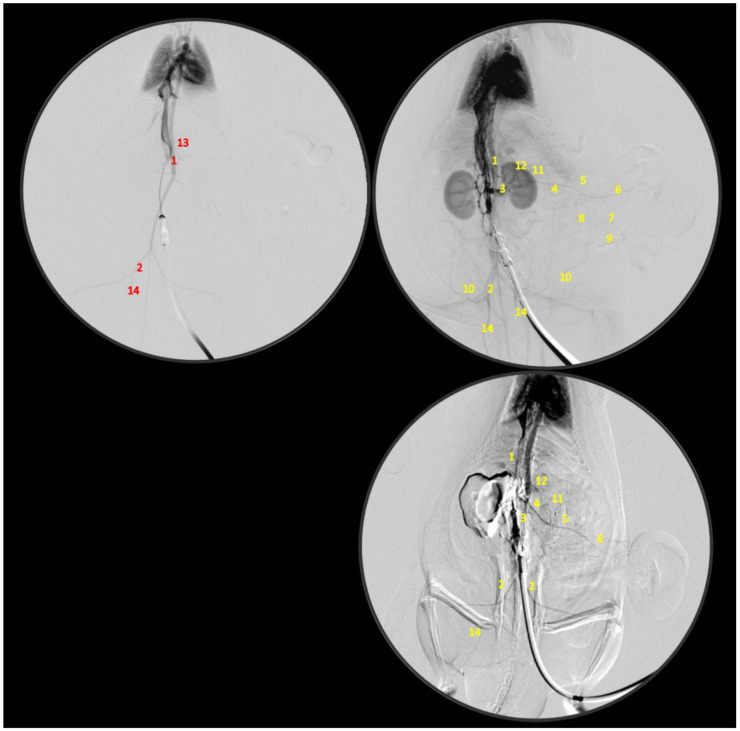
Inferior caval vein venography, and vessels presentation (abdominal aorta (1), common iliac artery (2), renal artery (3), superior mesenteric artery distal to occlusion (4), jejunal branches of superior mesenteric artery (5), ileal branches of superior mesenteric artery (6), ileocolic-right colic truncus (7), middle colic artery (8), ileocolic artery (9), caudal epigastric artery (10), pancreaticoduodenal artery (11), coeliac truncus (12), superior mesenteric artery proximal to occlusion (13), internal iliac artery (14)) in rats with the occlusion of the superior mesenteric vein and artery immediately following medication (BPC 157 10 µg/kg (upper), or 10 ng/kg (low) (right) or saline 5 mL/kg (left)) given intraperitoneally at 15 min ligation-time.

**Figure 7:**
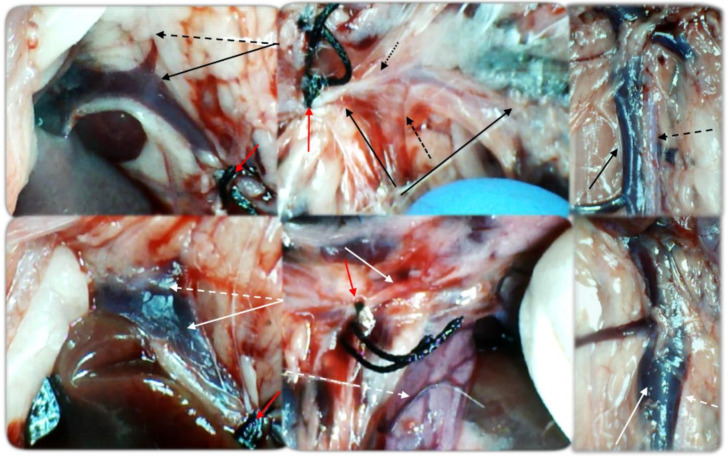
Illustrative gross vessel presentation in the rats with the occluded superior mesenteric vein and artery, without therapy (low, control) and BPC 157 therapy (upper, 10 ng/kg, providing the same presentation with the 10 µg/kg). Left. Pyloric vein (dashed arrow (black (BPC 157), white (control)) inflow into the portal vein (full arrow (black (BPC 157), white (control)), proximal to occlusion of the end of the superior mesenteric vein (red arrow). Reestablished blood flow (upper, BPC 157), congestion, and vascular failure (low, control). Middle. Superior mesenteric artery (full arrow (black (BPC 157), white (control)) inflow into the portal vein (full arrow (black (BPC 157), white (control)), proximal to occlusion and distal from occlusion (red arrow). Congested and tortuous vein of the ascending colon (white dashed arrow). Dashed black arrows indicate collaterals (big dashed arrow (medial colic artery), small dashed arrow (inferior anterior pancreaticoduodenal artery). Reestablished blood flow to part of superior mesenteric artery distal from occlusion filled with blood (upper, BPC 157), vascular failure, and empty superior mesenteric artery (low, control). Right. Inferior caval vein and abdominal aorta with presentation close to normal (black arrows (full (inferior caval vein), dashed (abdominal aorta), and congested inferior caval vein (full white arrow), and tinny empty abdominal aorta (dashed white arrow). Camera attached to a VMS-004 Discovery Deluxe USB microscope (Veho, Dayton, OH, USA).

**Figure 8:**
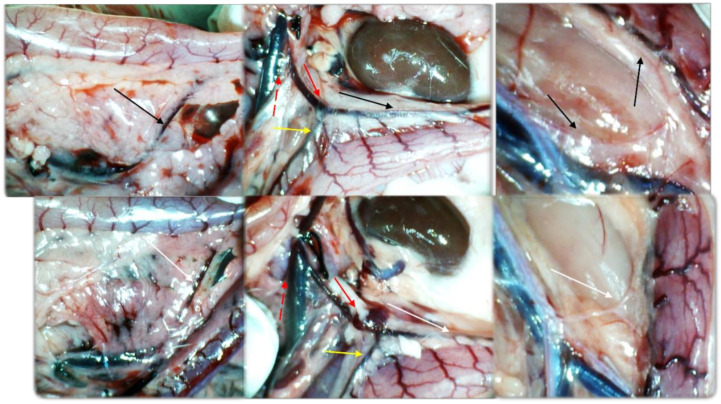
Illustrative gross vessel presentation in the rats with the occluded superior mesenteric vein and artery, without therapy (low, control) and BPC 157 therapy (upper, 10 ng/kg, providing the same presentation with the 10 µg/kg). Left. Inferior anterior pancreaticoduodenal vein (dashed arrow (black (BPC 157), white (control)) with prominent duodenal congestion (low, control). Reestablished blood flow (upper, BPC 157), congestion, and vascular failure (low, control). Middle. Superior mesenteric vein (red dashed arrows), inferior mesenteric vein (full red arrows), middle colic vein (yellow arrows), left colic vein (black (BPC 157), white (control) distal from occlusion. Tinny veins of the transverse colon (low, control), and exaggerated veins indicating activated collaterals (BPC 157) as a part of the reestablished blood flow to compensate for occlusion (upper, BPC 157). Right. Inferior mesenteric artery and abdominal aorta with presentation close to normal (black arrows (BPC 157), or tinny empty inferior mesenteric artery and abdominal aorta (low, control). Tortuous veins presentation of the rectal veins (control, low). Camera attached to a VMS-004 Discovery Deluxe USB microscope (Veho, Dayton, OH, USA).

**Figure 9:**
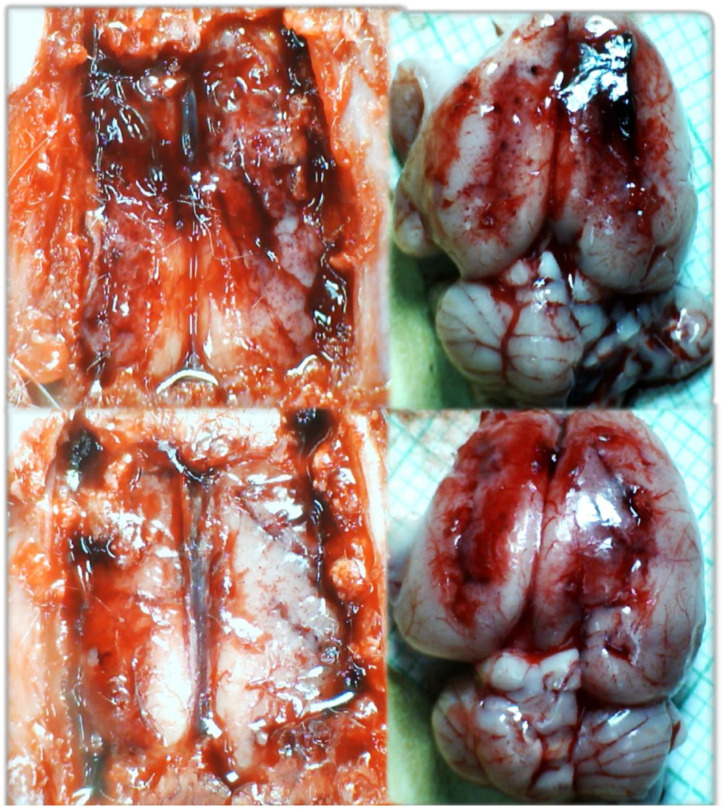
Illustrative brain presentation in the rats with the occluded superior mesenteric vein and artery, in the calvarial window immediately after vessels occlusion, and subsequent medication saline (5 mL/kg ip) (low, control) or BPC 157 (10 ng/kg ip) (upper)) (left) and after sacrifice (right), at the 30 min ligation-time. Saline (5 mL/kg ip) (low, control) or BPC 157 (10 ng/kg ip) (upper). Prominent brain swelling in control rats (low), completely reversed in BPC 157 rats (upper). Camera attached to a VMS-004 Discovery Deluxe USB microscope (Veho, Dayton, OH, USA).

**Figure 10:**
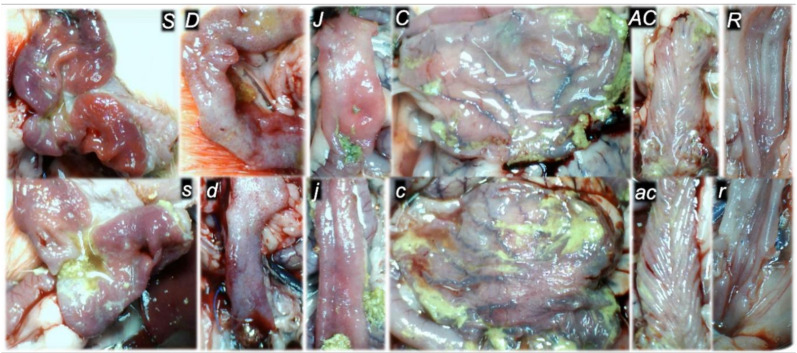
At 30 min ligation-time, gross presentation of the mucosa of the stomach (**s**,**S**), duodenum (**d**,**D**), jejunum (**j**,**J**), cecum (**c**,**C**), ascending colon (**ac**,**AC**), and rectum (**r**,**R**), in the rats with the occluded superior mesenteric vein and artery, in the rats that received saline medication (5 mL/kg ip) (control, low, small letters) or BPC 157 medication (10 ng/kg ip) (upper, capitals). Camera attached to a VMS-004 Discovery Deluxe USB microscope (Veho, Dayton, OH, USA).

**Figure 11:**
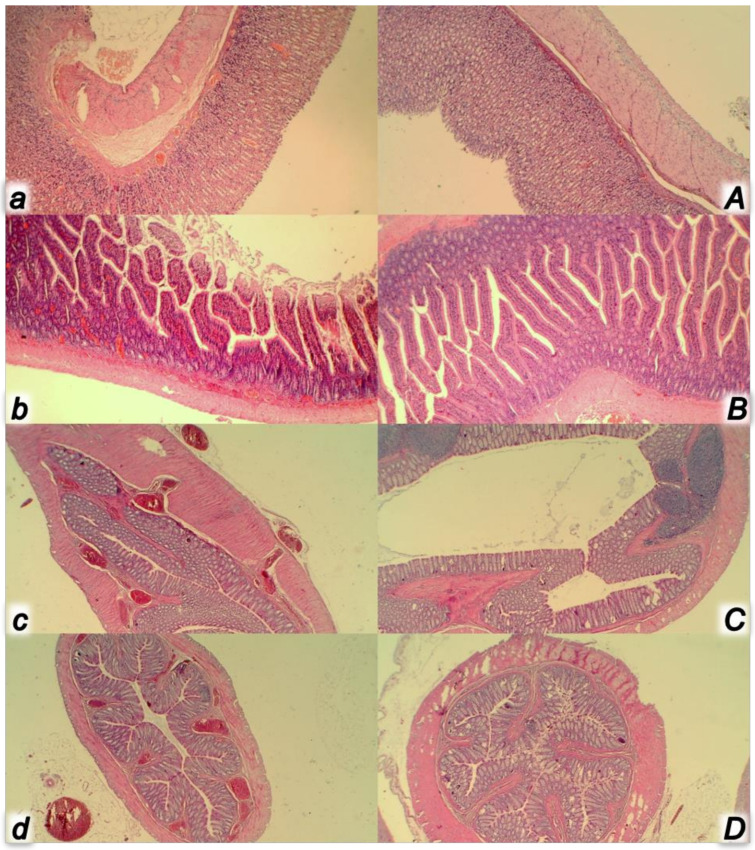
At 30 min ligation-time, microscopy presentation of the congestion of the stomach (**a**,**A**) and small intestine mucosa (**b**,**B**) and rectum (**c**,**C**) and perirectal vascular plexus (**d**,**D**) (HE, ×100) in the rats with occluded superior mesenteric vein and artery, controls (small letters) and BPC 157 treated (capitals). Control rats with occluded superior mesenteric vein and artery exhibited the congestion and dilatation of the small vessels in the stomach (**a**) and small intestine mucosa (**b**), congestion and dilatation of the small vessels of the rectum wall (**c**) along with congestion of perirectal blood vessels (**d**). Contrarily, BPC 157 treated rats showed no changes in the stomach (**A**) and small intestine mucosa (**B**) and no changes in the rectum (**C**) and perirectal vascular plexus (**D**).

**Figure 12:**
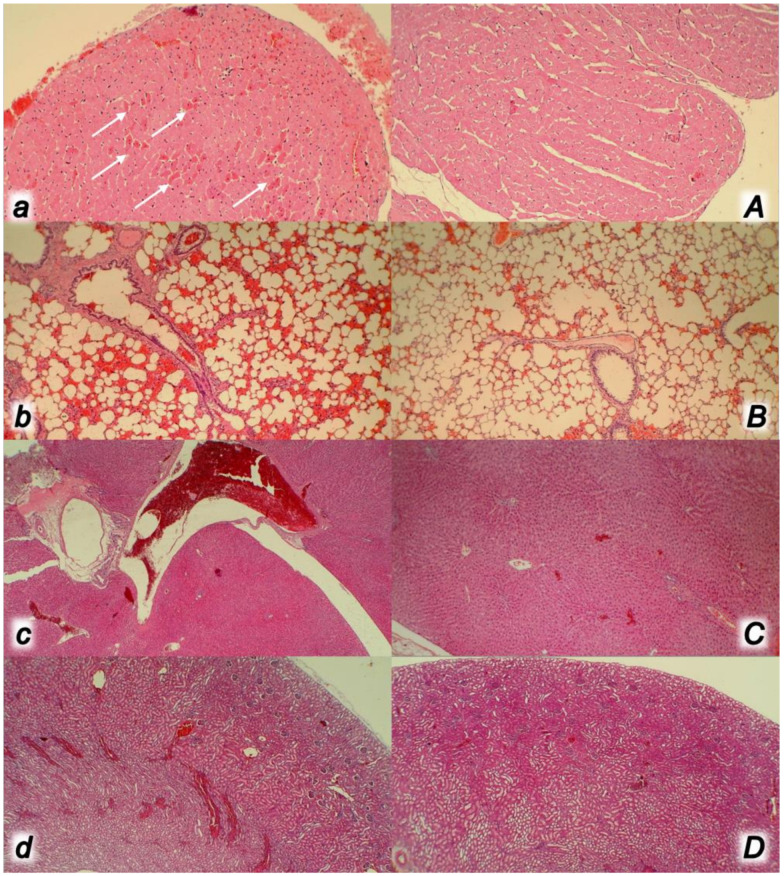
At 30 min ligation-time, microscopy presentation of the congestion of the heart (**a**,**A**) (HE, ×200), lung (**b**,**B**) (HE, ×100), liver (**c**,**C**) (HE, ×100), and the renal parenchyma (**d**,**D**) (HE, ×100) in the rats with occluded superior mesenteric vein and artery, controls (small letters) and BPC 157 treated (capitals). Control rats with occluded superior mesenteric vein and artery exhibited the subendocardial infarct as well as congestion within the myocardium (**a**) and lung septa along with intra-alveolar hemorrhage (**b**), liver congestion and dilatation of sinusoids, portal vein, and central veins (**c**) along with congestion of renal parenchyma and glomeruli (**d**). BPC 157 treated rats showed no heart congestion and heart infarct (**A**) along with minimal lung congestion and intra-alveolar hemorrhage (**B**) and no changes in the liver (**C**) and renal parenchyma (**D**).

**Figure 13:**
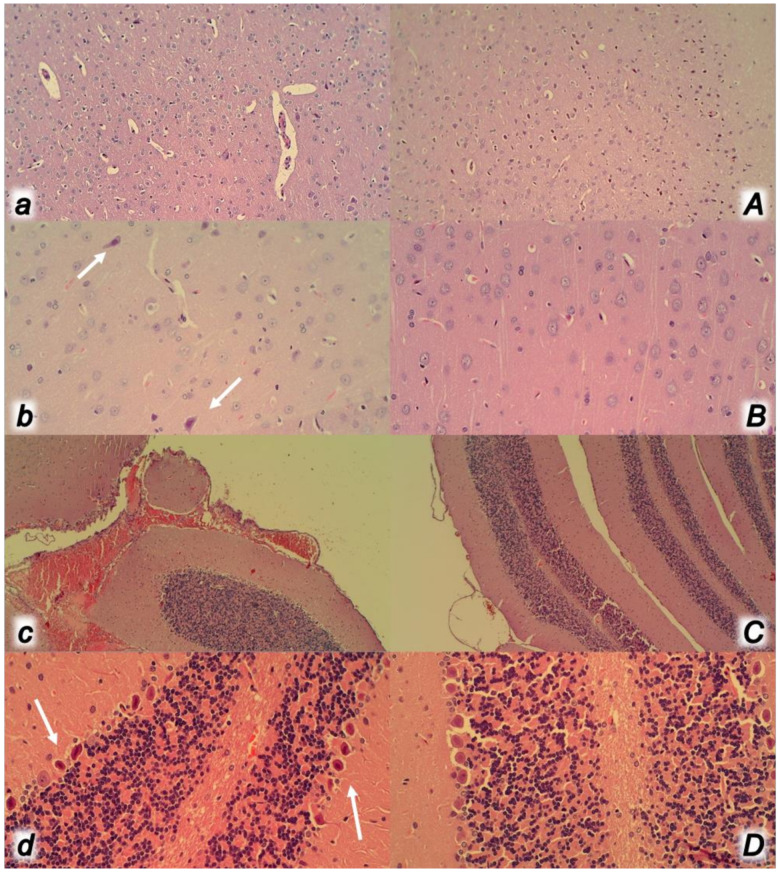
At 30 min ligation-time, microscopy presentation of the neuropathologic changes in the cerebral cortex (**a**,**A** (HE, ×200), **b**,**B** (HE, ×400)) and cerebellar cortex (**c**,**C** (HE, ×100), **d**,**D** (HE, ×400)) areas of the rats with occluded superior mesenteric vein and artery, controls (small letters) and BPC 157 treated (capitals). Cerebral cortex (**a**,**A**,**b**,**B**). While control rats with occluded superior mesenteric vein and artery exhibited marked edema and congestion (**a**), BPC 157 treated rats presented only mild edema (**A**). A scant karyopyknosis presented control rats with occluded superior mesenteric vein and artery in the cerebral cortex (arrows) (**b**) while no changes appeared in BPC 157 treated rats (**B)**. Cerebellar cortex (**c**,**C**,**d**,**D**). Control rats with occluded superior mesenteric vein and artery exhibited subarachnoid hemorrhage at the base of the brain in the cerebellar area (**c**) and multiple karyopyknosis of Purkinje cells of the cerebellar cortex (arrows) (**d**), unlike BPC 157 treated rats which remained without change (**C**,**D**).

**Figure 14:**
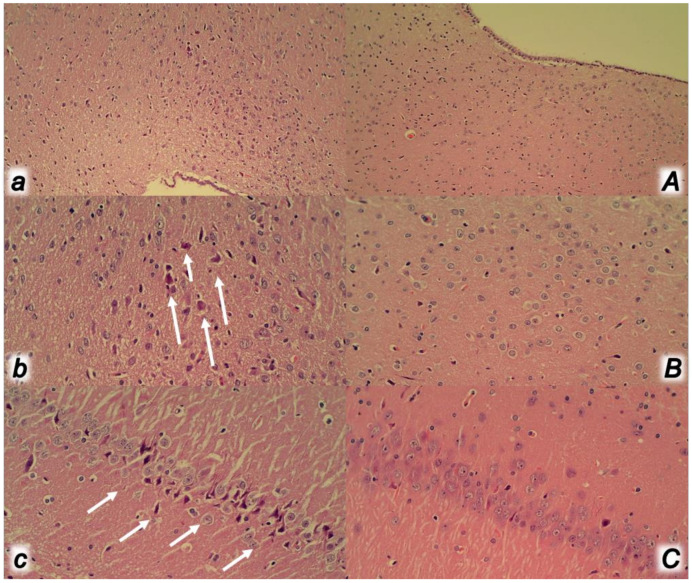
At 30 min ligation-time, microscopy presentation of the neuropathologic changes in the hypothalamus (**a**,**A** (HE, ×100), **b**,**B** (HE, ×200)) and hippocampus (**c**,**C** (HE, ×200)) in the rats with occluded superior mesenteric vein and artery, controls (small letters) and BPC 157 treated (capitals). Control rats with occluded superior mesenteric vein and artery exhibited in the hypothalamus and hippocampus edema (**a**,**c**) and karyopyknosis of the hypothalamic (arrows) (**b**) and hippocampal (arrows) (**c**) cells; only minimal hypothalamic edema in BPC 157 (**A**) and no karyopyknosis of the hypothalamic cells (**B**), and no changes in the hippocampus (**C**).

Simultaneous occlusion of the superior mesenteric vein and artery (failed flow through the superior mesenteric vein and artery and their tributaries) produced a marked noxious syndrome (Figure 1, Figure 2, Figure 3, Figure 4, Figure 5, Figure 6, Figure 7, Figure 8, Figure 9, Figure 10, Figure 11, Figure 12, Figure 13 and Figure 14). Application of BPC 157 in the rats with an occluded superior mesenteric vein and artery had a considerable therapeutic effect. Unlike controls, in all BPC 157–treated rats, there was simultaneous activation of venous and arterial collateral pathways (Figure 5, Figure 6, Figure 7 and Figure 8; Fisher’s exact test *p* ˂ 0.05). Angiography and direct recording demonstrated activation of the superior mesenteric vein–inferior anterior pancreaticoduodenal vein–superior anterior pancreaticoduodenal vein–pyloric vein–portal vein pathway, an alternative pathway towards the inferior caval vein via the united middle colic vein and inferior mesenteric vein through the left colic vein, and the inferior anterior pancreaticoduodenal artery and inferior mesenteric artery (Figure 5, Figure 6, Figure 7 and Figure 8).

BPC 157 treatment counteracted blood pressure disturbances centrally (superior sagittal sinus hypertension) and peripherally (portal and caval hypertension, aortal hypotension). BPC 157 eliminated portal and caval hypertension and markedly ameliorated aortal hypotension and superior sagittal sinus hypertension. It should be noted that considering the markedly negative pressure values in the superior sagittal sinus in normal rats, BPC 157 induced, in the superior sagittal sinus, the needed reversal of the excessively positive values to the normal negative values (Figure 1). BPC 157 also counteracted thrombosis centrally (superior sagittal sinus) and peripherally in veins (portal vein and inferior caval vein, which had the largest clot formation, superior mesenteric vein, hepatic veins, and external jugular vein) as well as in arteries (superior mesenteric artery and abdominal aorta) (Figure 1).

BPC 157 had notable effects on the relative volume of the arteries, veins, and brain (Figure 1). These findings apparently underscore the counteraction of the blood stasis by activation of the collateral pathways to compensate for major vessel occlusion (Figure 5, Figure 6, Figure 7 and Figure 8). BPC 157 decreased the relative volume of the superior mesenteric artery proximal to the ligation and increased the relative volume distal to the occlusion (Figure 1, as seen with the filled superior mesenteric artery both directly and with camera recording in Figure 6) as well as by venography (Figure 8). Likewise, BPC 157 increased the relative volume of the inferior mesenteric artery and abdominal aorta (Figure 1), which appeared to be empty in rats in which the superior mesenteric vein and artery had been occluded (as a part of the reorganised blood flow, Figure 8). BPC 157 decreased the relative volume of the portal, pyloric, inferior caval, and inferior anterior pancreaticoduodenal veins (Figure 1). These veins appeared congested (Figure 7 and Figure 8), likely due to the trapped blood volume; congestion was counteracted by the activation of the collateral bridging pathway, and a connection between the superior mesenteric vein and portal vein was re-established (Figure 5). BPC 157 increased the relative volume of the inferior mesenteric vein, middle colic vein, and left colic vein (Figure 1). These veins, which appeared tiny (Figure 8), became filled with blood upon BPC 157 administration as a part of the reorganised blood flow to compensate for the occlusion of the end of the superior mesenteric vein, from the superior mesenteric vein towards the rectal, left iliac, and inferior caval veins. Finally, BPC 157 induced a rapid and considerable decrease in brain swelling and reduced brain volume (Figure 1 and Figure 9).

Alongside the aforementioned findings, BPC 157 counteracted the ECG disturbances in the rats with simultaneous occlusion of the superior mesenteric vein and artery, namely severe tachycardia and peaked P waves, prolonged PQ, and QTc intervals, and ST elevation (Figure 2). In addition, heart sections presented normal histology, unlike the subendocardial infarction seen in controls (Figure 12).

BPC 157 also counteracted the ongoing oxidative stress in the cecum; the elevated MDA level in the rats with simultaneous occlusion of the superior mesenteric vein and artery was reduced to control levels after BPC 157 administration (Figure 2).

BPC 157 counteracted gastrointestinal lesions that appeared in the untreated rats with simultaneous occlusion of the superior mesenteric vein and artery (Figure 3), which presented increased severity from the upper towards the lower portion of the (Figure 10 and Figure 11). In control rats with simultaneous occlusion of the superior mesenteric vein and artery, the tissue damage increased from the stomach to the large bowel, with marked transmural congestion within the stomach, duodenum, intestine, and rectum (Figure 11). In the stomach, dilated capillaries in the lamina propria appeared. Within the small and large bowel mucosa, focal haemorrhage in the lamina propria emerged with moderate mucosal injury and blunt intestinal villi (Figure 11). Even more severe congestion appeared in the rectum along with congestion of perirectal vascular plexus (Figure 11) (likely as part of the increased stasis providing the increased clot formation in the close inferior caval vein). These findings are consistent with the serosal disturbances (increased congestion and failed arterial failing and ramification) (Figure 4). On the contrary, most of these changes were not present in rats treated with BPC 157 after simultaneous occlusion of the mesenteric vein and artery (Figure 10 and Figure 11).

Without BPC 157 therapy, the rats with the occluded superior mesenteric vein and artery showed considerable lesions in the lungs, liver, kidneys, and heart (Figure 12). In the renal parenchyma, there was mild degeneration of proximal tubules, severe vascular congestion, and mild interstitial oedema (Figure 12). The liver parenchyma exhibited mild activation of Kupffer cells and severe dilatation of sinusoids with liver congestion (Figure 12). Severe congestion occurred within the myocardium along with the subendocardial infarct (Figure 12). Focal thickening of the alveolar membranes occurred alongside lung congestion, pulmonary oedema, marked intra-alveolar haemorrhage, and focal interstitial neutrophil infiltration (Figure 12). Rats treated with BPC 157 exhibited no lesions in the kidney, liver, and heart, and showed only discrete lung oedema, congestion, and intra-alveolar haemorrhage with no other changes in the lung parenchyma (Figure 3 and Figure 12).

Without therapy, the rats with simultaneous occlusion of the superior mesenteric vein and artery exhibited subarachnoid haemorrhage at the base of the brain in the cerebellar area and more karyopyknotic cells in the cerebral and cerebellar cortex, hippocampus, and hypothalamus/thalamus (Figure 13 and Figure 14). Neuropathologic changes in cerebral cortical areas revealed increased oedema and congestion (Figure 13). There were karyopyknosis and degeneration of Purkinje cells of the cerebellar cortex and pyramidal cells of the hippocampus (Figure 13 and Figure 14). By contrast, along with other beneficial effects (especially the reversed superior sagittal sinus hypertension, Figure 1), rats treated with BPC 157 showed only a few karyopyknotic neuronal cells in the analysed neuroanatomic structures, primarily in the hippocampus, and did not haemorrhage (Figure 13 and Figure 14).

BPC 157 administration led to intriguing findings with venography (1 mL delivered over 30 s in the superior mesenteric vein or inferior caval vein) after 15 min of ligation when there was already an advanced noxious course (Figure 5 and Figure 6). Control rats with the ligated superior mesenteric vein and artery regularly showed a poor presentation (Figure 5), and they responded with rapid rupture of the superior mesenteric vein or without a sign of the superior mesenteric artery. The rats treated with BPC 157 presented activation of the collateral pathways, a re-established superior mesenteric vein–portal vein connection, and refilled superior mesenteric artery distal to the occlusion. Since these effects can be rapidly induced even at the advanced stage of the injury course, the ability of BPC 157 to counteract advanced disturbances should be emphasized.

Serum ALT and AST values increased in control rats with simultaneous occlusion of the superior mesenteric vein and artery, and BPC 157 administration reduced these values (Figure 3). Likewise, in the rats with the occluded superior mesenteric vein and artery, we noted an increase in the liver and spleen relative weight, which was markedly counteracted after BPC 157 therapy (Figure 3).

In summary, BPC 157 therapy in rats with simultaneous occlusion of the superior mesenteric vein and artery provided marked benefits: there was a reversal of portal and caval hypertension, aortal hypotension, and superior sagittal sinus hypertension; there was markedly attenuated arterial and venous thrombosis centrally and peripherally; and there was the elimination of brain, heart, lung, liver, kidney, and gastrointestinal lesions. These counteractions were ascribed to the simultaneous activation of particular venous and arterial collateral pathways located centrally and peripherally.

## 4. Discussion

Simultaneous occlusion of the superior mesenteric artery and vein is a rare, serious complication that is hardly ever resolved [1,2]. Hence, it requires a novel therapeutic approach, including BPC 157, which could alleviate venous occlusion syndromes by activating bypass pathways [5,6,7,8,9,10,11,12]. Since BPC 157 is an effective therapy in rats with peripheral venous occlusion syndromes [5,6,7], we tested whether it could also counteract the simultaneous occlusion of the end of the superior mesenteric vein and the superior mesenteric artery close to its aortal origin. That activated ‘bypassing key’ involves the superior mesenteric vein–inferior anterior pancreaticoduodenal vein–superior anterior pancreaticoduodenal vein–pyloric vein–portal vein pathway, an alternative pathway towards the inferior caval vein via the united middle colic vein and inferior mesenteric vein through the left colic vein, and the inferior anterior pancreaticoduodenal artery and inferior mesenteric artery. Thus, consistently with the benefits of BPC 157 in venous occlusion syndromes [5,6,7], we have revealed the counteraction potential of this peptide when there is simultaneous occlusion of the superior mesenteric artery and vein. BPC 157 attenuated/eliminated the superior sagittal sinus, portal and caval hypertension, aortal hypotension, thrombosis, and organ lesions that occurred peripherally and centrally, and ECG disturbances. The full abrogation of the syndrome, as had been shown with venous occlusion syndromes [5,6,7], may suggest there was simultaneous activation of the arterial and venous pathways, leading to the rapid regain of the superior mesenteric vein and portal vein pathway and the superior mesenteric artery supply, to compensate the arterial and venous flow despite the permanent obstruction. The complete and simultaneous obstruction of the mesenteric vein and artery was instant and thereby provides no time for adequate spontaneous adaptation unless an essential therapy is active. Taken together, these results suggest that BPC 157 therapy is effective for rats with permanent occlusion of the superior mesenteric vein and artery.

We argue that we have provided a proof of principle that BPC 157 ameliorates multiorgan failure syndrome based on the elimination of severe portal and caval hypertension and aortal hypotension and the increased pressure in the superior sagittal sinus as well as the rapid collateral vessel recruitment regardless of the precise mechanism [3,4]. Of note, it remains to be fully specified how ‘endothelium maintenance → epithelium maintenance’ may go to the advanced ‘endothelium maintenance → epithelium maintenance = blood vessel recruitment and activation’ (‘running’) towards the site of injury, also described as ‘bypassing’ the occlusion via alternative pathways and to resolving the simultaneous permanent occlusion of a vein and artery. As mentioned previously, endothelium protection may be immediate, as shown with the maintenance of endothelium function to counteract stomach mucosal lesions when exposed to absolute alcohol [20,30], combined with a direct effect on vasomotor tone [29] and ischemia-induced increased capillary permeability [37]. Part of this defensive pathway, which needs to be fully determined, may be the already mentioned BPC 157 effects: activation of the Src–caveolin-1–eNOS pathway [29], activation of the VEGFR2–Akt–eNOS signalling pathway without the need of other known ligands or shear stress [33] and membrane stabilisation [37]. Counteracting leaky gut syndrome, via increased tight junction protein ZO-1 expression and transepithelial resistance [37], would avoid a shift of fluid and proteins from the intravascular to the extravascular space and thus prevent an increase in intra-abdominal pressure [27]. Due to the interaction among the three body cavities, the increased intra-abdominal pressure leads to increased intracranial pressure and vice versa [27]. The fact that BPC 157 can inhibit the mRNA expression of inflammatory mediators (iNOS, IL-6, IFNγ, and TNF-α) as well as increase protein expression of HSP70, HSP90 and promote the expression of antioxidant proteins, such as HO-1, NQO-1, glutathione reductase, glutathione peroxidase 2 and GST-pi [37] indicate that this peptide interacts with several molecular pathways [5,25,29,31,32,33,34,35,36,37]. BPC 157 also has a modulatory role on the NO system and NO agents [3,4,28,30], in particular, on blood pressure [30] and thrombocyte function [22,23,24]. Indeed, BPC 157 counteracts hypertension and pro-thrombotic effects induced by the NOS blocker L-NAME as well as the hypotension and anti-thrombotic effects induced by the NOS substrate L-arginine [24,30]. In addition, BPC 157 may induce NO release [30,47]. Likewise, BPC 157 interacts with the prostaglandin system and may counteract gastrointestinal, liver, brain, and bleeding disorders that may be induced with NSAIDs and non-specific and specific agents [37,48].

Similar to the previous studies with venous occlusion [5,6,7,8,9,10,11,12], there were signs that BPC 157 attenuated the Virchow triad—thrombosis in all vessels investigated (veins and arteries) peripherally and centrally—and thereby counteracted stasis. Superior mesenteric vein and artery obstruction would immediately create a complex multiorgan dysfunction syndrome; consequently, effective therapy would require coordinated benefits on the affected target organs. Rats with an occluded superior mesenteric vein and artery may quickly experience a lack of circulation, as has been seen in rats with inferior caval vein syndrome, Pringle manoeuvre ischemia/reperfusion, and Budd–Chiari syndrome [5,6,7]. This may induce a generalised vessel failure, stasis, and thrombosis peripherally and centrally as well as heart failure. Thereby, lung congestion, liver and kidney failure (substantial congestion of central vein as well as branches of portal veins in portal triads, inferior caval vein, and superior mesenteric vein congestion), prominent portal and caval hypertension, aortal hypotension, and gastrointestinal haemorrhagic lesion, with an ascending sequence from the stomach to the large bowel tissue damage can appear [5,6,7]. Elevated superior sagittal sinus pressure and brain lesions result from the inability to drain venous blood adequately for a given cerebral blood inflow. Such venous and intracranial hypertension or increased intra-abdominal (and intra-thoracic) pressure are rapidly transmitted through the venous system and increase intracranial pressure [49,50,51,52]. On the other hand, for the circulation under the barriers of the obstruction points, the activated ‘bypassing key’ may be the adequate defensive response at any time (note, angiography findings demonstrated BPC 157 therapy effects even after prolonged arterial and venous occlusion).

The central effects induced by BPC 157—reduced gross brain swelling and markedly counteracted brain lesions and superior sagittal sinus hypertension—also underscore the benefits of this therapy. Furthermore, BPC 157 has been shown to counteract doxorubicin-induced chronic heart failure [53], various arrhythmias induced by different agents, and noxious procedures [54,55,56,57,58,59], including prolonged QT intervals [56], even those which are centrally mediated. Likewise, researchers have shown that BPC 157 counteracts the various severe lesions in the lung [60,61,62], liver [63,64,65,66,67,68,69,70], and gastrointestinal tract [65,66,67,68,69,70], particularly those that were induced rapidly. Moreover, the reduced MDA and preservation and rescue of intestinal mucosal and vessel integrity may be the result of the free radical scavenger activity of BPC 157 to counteract reperfusion-induced oxidative injury in the colon, duodenum, cecum, liver, veins, and plasma [5,6,8,9,10,11,12,36,37,38,39,40]. Thus, we speculate that BPC 157 may counteract several steps of the syndrome that results from vessel occlusion. Finally, BPC 157 has been shown to attenuate brain lesions induced by trauma [71], spinal cord compression [72], or various encephalopathies [65,66,67,68,69,70]. BPC 157 also counteracted both early and delayed neural hippocampal damage in a rat stroke model (bilateral occlusion of the common carotid arteries) when therapy was given during reperfusion, leading to full functional recovery and restoration of recognition memory deficits [25]. BPC 157 appears to regulate the expression of several genes: strongly elevated *Egr1*, *Akt1*, *Kras*, *Src*, *Foxo*, *Srf*, *Vegfr2*, *Nos3,* and *Nos1* and decreased *Nos2* and *Nfkb* (*Mapk1* not activated); these findings can be followed up to determine the exact mechanism by which BPC 157 exerts its benefits [25].

In conclusion, our findings provide evidence that the stable gastric pentadecapeptide BPC 157 may participate, peripherally and centrally, in resolving the consequences of vascular occlusion and may abrogate the noxious effects throughout the body [3,4,5,6,7,8,9,10,11,12]. Our study should overcome the discouraging point that animal studies per se should be interpreted with caution and the relative paucity of BPC 157 clinical data [3,4]. Namely, BPC 157 has been shown to be efficacious in ulcerative colitis, both in clinical settings [73,74] as well as in experimental rat models of ischemia/reperfusion vascular ulcerative colitis [11] and other ulcerative colitis models [37,65,66,67,68,69,70]. A particular encouraging point is that BPC 157 has a very safe profile (lethal dose could not be achieved) [3,4,13], a point recently confirmed in a large study [75]. In this context, animal models are indispensable to ensure the efficacy of BPC 157. Thus, while additional studies need to be done, we could claim that BPC 157 therapy rapidly overcomes the simultaneous occlusion of the superior mesenteric artery and vein and consequent noxious syndrome in rats. This finding also emphasises the noted bypassing principle in arterial and venous occlusion [5,6,7,8,9,10,11,12]. Rapidly activating alternative bypassing pathways overrides the permanent ligation of the superior mesenteric artery and vein and, hopefully, may indicate the therapeutic strategies to compensate simultaneously for occlusion of the artery and vein.

## Figures and Tables

**Table 1:** The neuropathologic scores.

Brain Area	Grading	Percent Area Affected	Morphological Changes
Cerebral and cerebellar cortex, hypothalamus, thalamus, hippocampus	1	≤10	Small, patchy, complete or incomplete infarcts
	2	20–30	Partly confluent complete or incomplete infarcts
	3	40–60	Large confluent compete infarcts
	4	>75	In cortex; total disintegration of the tissue, in hypothalamus, thalamus, hippocampus; large complete infarcts
Cerebral and cerebellar cortex, hypothalamus, thalamus, hippocampus	1	≤20	A few karyopyknotic of neuronal cells
	2	50	Patchy areas of karyopyknotic areas
	3	75	More extensive of karyopyknotic areas
	4	100	Complete infarction

## Data Availability

The data presented in this study are available on request from the corresponding author.
